# Supplementary material for: Atomic-scale imaging of emergent order at a magnetic field–induced Lifshitz transition
Source: Sci Adv. 2022 Sep 30;8(39):eabo7757. doi: 10.1126/sciadv.abo7757 (PMC9524824; doi:10.1126/sciadv.abo7757)
Supplement: Supplementary file 1 — Sections S1 to S10 Figs. S1 to S12 Table S1 References [file sciadv.abo7757_sm.pdf]

Science Advances
AAAS

Supplementary Materials for

Atomic-scale imaging of emergent order at a magnetic field–induced Lifshitz transition

Carolina A. Marques *et al.*

Corresponding author: Peter Wahl, wahl@st-andrews.ac.uk

Sci. Adv. **8**, eabo7757 (2022)
DOI: 10.1126/sciadv.abo7757

This PDF file includes:

Sections S1 to S10
Figs. S1 to S12
Table S1
References

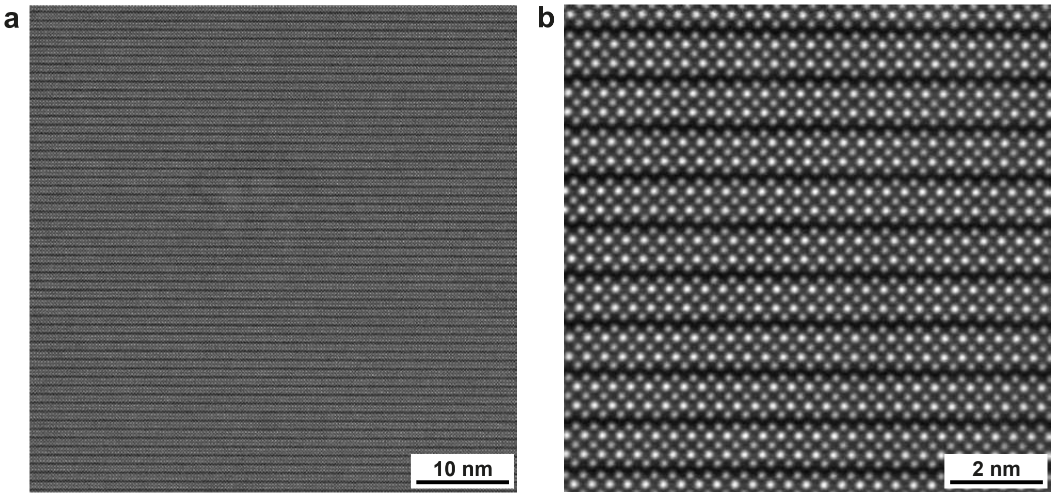

FIG. S1: **Transmission electron microscope images of $Sr_3Ru_2O_7$.** High-angle annular dark field (HAADF) images obtained form a lamella cut along the $\langle 1\,1\,0 \rangle$ direction from the $\langle 0\,1\,0 \rangle$ zone axis of $Sr_3Ru_2O_7$. **a**, A $59 \times 59\text{nm}^2$ field of view, showing no stacking faults for this crystal. **b**, A 10.5×10.5nm field of view, showing the bilayer stacking of the RuO_6 octahedra (brighter spots) and Sr atoms (darker spots).

S1. SAMPLE CHARACTERISATION

A. TEM

Figure S1(a) shows an atomic resolution TEM image of a cross section of a $Sr_3Ru_2O_7$ sample from the same batch as the ones on which we have performed STM. The image demonstrates the high quality of the single crystals, with high uniformity across the field of view. Figure S1(b) shows a higher magnification image with atomic resolution, with the expected bilayer structure of $Sr_3Ru_2O_7$ and no evidence for inclusions of other members of the Ruddlesden-Popper series. The image shows the Ru atoms as bright spots, and the Sr atoms slightly darker.

B. Transport and thermodynamic measurements

Figure S2(a) shows the temperature dependence of the resistivity of a sample of the same batch as the samples measured in STM, from room temperature to 2K. The residual-resistiviy ratio obtained is $R(300\text{K})/R(2\text{K}) \sim 102$, consistent with high quality samples. Fitting the expression $\rho(T) = \rho_{\text{res}} + AT^{\alpha}$ to the low temperature regime (inset), gives a residual resistivity of $\rho_{\text{res}} = 1.3\mu\Omega\text{cm}$. Figure S2(b) shows resistivity curves as a function of temperature in magnetic fields up to 9T, where the slope at low temperatures (see inset) is seen to change close to fields of 8T. The fit of $\rho(T)$ to the low temperature part of the resistivity curve at 1T, Figure S2(c), shows a coefficient $\alpha = 2$, consistent with Fermi liquid behaviour. At 8T, the fit of $\rho(T)$ (Figure S2(d)) gives $\alpha = 1$, as expected in the vicinity of the metamagnetic transition of $Sr_3Ru_2O_7$[4]. The result of fitting $\rho(T)$ to the resistivity curves taken in different magnetic fields is shown in Figure S3(a), where the color plot shows the exponent α as a function of temperature and magnetic field. Around 8T, α is seen to change to 1, which is the typical signature found in $Sr_3Ru_2O_7$ near the quantum critical end point[4]. Figure S3(b), shows a specific heat curve at 0T from a sample of the same batch, where the characteristic broad peak below $\sim$ 20K is observed.

S2. PROCESSING OF STM DATA

To remove artifacts in our data which result from the measurement process, and optimize the experimental parameters, we have removed drift due to hysteresis of the scan piezo over the course of a measurement, and used unfolding of the Fourier transformation as described in the following.

A. Correction of piezo hysteresis and drift

During acquisition of topographic images and spectroscopic maps, the position of the STM tip is affected by hysteresis of the piezo response and piezo creep, inducing drifts in

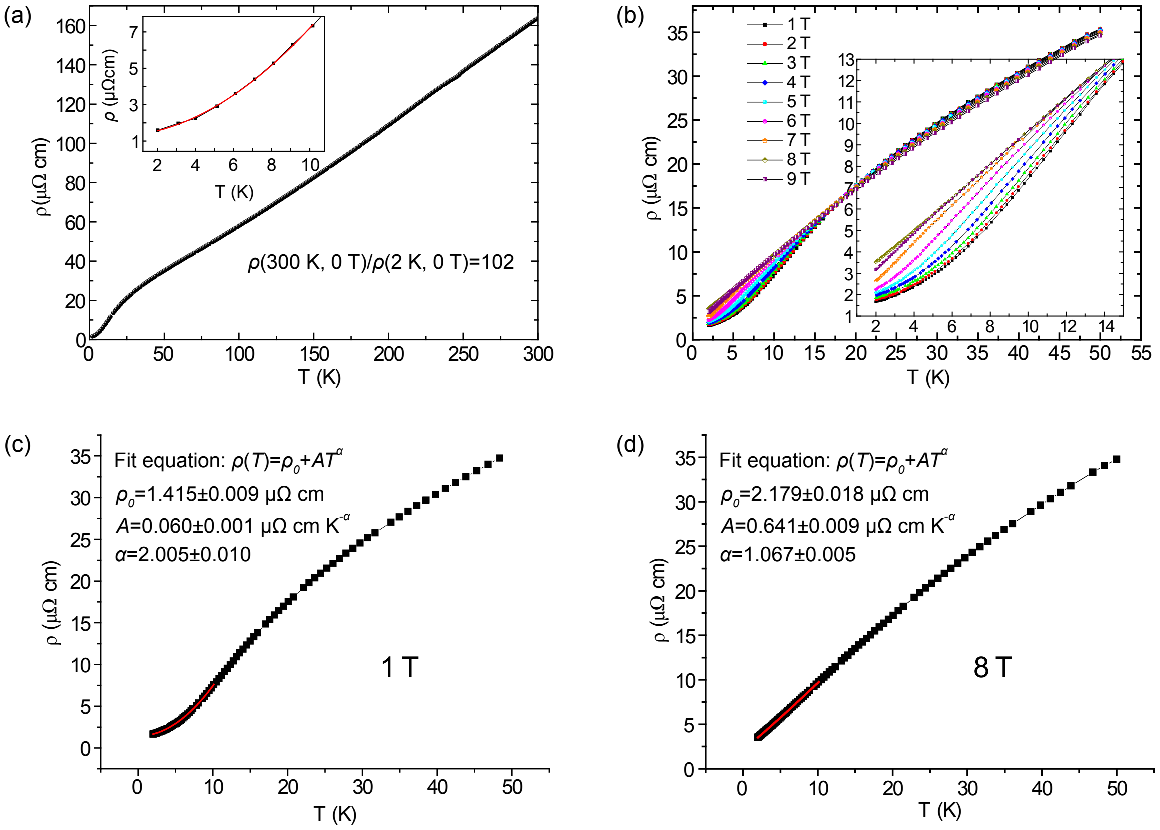

FIG. S2: **Bulk resistivity of $Sr_3Ru_2O_7$. a**, Resistivity as a function of temperature, from room temperature to 2K. We obtain a residual-resistivity ratio $R(300\text{K})/R(2\text{K}) \sim 102$, confirming the high quality of the samples. The inset shows a fit of the function $\rho(T, B) = AT^{\alpha} + \rho_{\text{res}}$ to the low temperature data, giving $\rho_{\text{res}} = 1.3\mu\Omega\text{cm}$. **b**, Resistivity at different magnetic fields, from 0T to 9T. The inset shows a close up of the low temperature data. In proximity of the metamagnetic transition, a clear change in slope of the resistivity at low temperatures is seen. **c**, Resistivity curve at 1T with a fit of $\rho(T)$ at temperatures $T = 0\ldots10\text{K}$, showing an exponent $\alpha = 2$ as expected for Fermi liquid behaviour. **d**, Resistivity curve at 8T with a fit of $\rho(T)$ as in **c** resulting in $\alpha = 1$, indicative of deviations from Fermi liquid behaviour close to the metamagnetic transition.

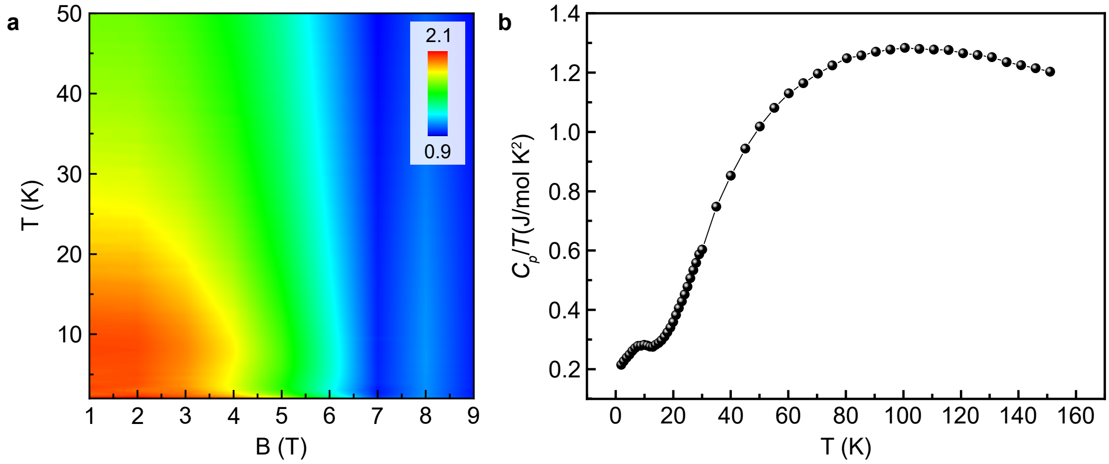

FIG. S3: **Transport and thermodynamic measurements of $Sr_3Ru_2O_7$ samples.** **a**. Exponent α of the resistivity $\rho(T,B) = AT^\alpha + C$ as a function of temperature T and magnetic field B across the metamagnetic transition. **b**. Specific heat at zero applied magnetic field, $B = 0$T, showing the characteristic broad maximum below 20K.

the position of the STM tip on the order of picometers resulting in small distortions of the image or map due to uncertainty in the tip position $\delta\mathbf{u}(\mathbf{r})$. $\delta\mathbf{u}(\mathbf{r})$ is expected to vary only slowly with time. The impact of this uncertainty can be most readily seen from the atomic lattice, which will not be a perfect square lattice anymore. As a consequence, while the Fourier transformation (FT) should show four delta-function like Bragg peaks at the corners of a perfect square, the two independent sets of Bragg peaks will typically not be at a 90° angle from each other, and not exactly exhibit the same magnitude $|\mathbf{q}_{\mathrm{at}}|$. Further, the Bragg peaks will not show up as delta-functions, they will have an intrinsic width due to variations of the periodicity across the image. To correct for these small variations, we apply a transformation to our image, mapping the distorted atomic lattice back onto a perfect square lattice. This transformation is determined by comparing the measured topographic image $z_{\mathrm{m}}(\mathbf{r})$ with the expected ideal square lattice, allowing to reconstruct a corrected $z(\mathbf{r})$ image. The transformation is determined using a lock-in algorithm[40] to

detect the displacement $\delta\mathbf{u}(\mathbf{r})$ required to locally map the image onto a perfect lattice. We note that this algorithm requires sufficient sampling of the atomic periodicity.

The perfect square lattice can be described by

$$T(\mathbf{r}) = \cos(\mathbf{q}_1 \cdot \mathbf{r} + \phi_1) + \cos(\mathbf{q}_2 \cdot \mathbf{r} + \phi_2), \tag{S1}$$

where $\mathbf{q}_1$ and $\mathbf{q}_2$ are the $\mathbf{q}$-vectors corresponding to the Bragg peaks ($|q_i| = 2\pi/a_{\mathrm{Sr}}$, where a_{Sr} is the lattice constant of the Sr square lattice), and ϕ_1 and ϕ_2 are phases which can be chosen to be zero. For simplicity, but without loss of generality, we consider $\mathbf{q}_1$ and $\mathbf{q}_2$ to be aligned with the x and y directions. For this, we rotate our measured image $z_{\mathrm{m}}(\mathbf{r})$ such that one of the Bragg peaks is aligned with the y-axis. We take $\phi_1 = \phi_2 = \phi$ as the phase of the Bragg peak in the y direction taken from the FT of the topography of the map. For the lock-in method, we implement phase-sensitive detection for each of the two independent spatial directions by calculating

$$\begin{aligned} X_i(\mathbf{r}) &= \mathcal{L}\left(z_{\mathrm{m}}(\mathbf{r})\cos(\mathbf{q}_i \cdot \mathbf{r}), \Lambda\right) \\ Y_i(\mathbf{r}) &= \mathcal{L}\left(-z_{\mathrm{m}}(\mathbf{r})\sin(\mathbf{q}_i \cdot \mathbf{r}), \Lambda\right). \end{aligned}$$

To enable the phase-sensitive detection and extract these slow variations of the phase, we apply a low-pass filter $\mathcal{L}$ with width $\Lambda \gg \frac{\pi}{|\mathbf{q}_i|}$. We obtain the quantities $X_i(\mathbf{r})$ and $Y_i(\mathbf{r})$ for the x- and y-directions which allow us to extract the phase $\Theta_i(\mathbf{r})$ associated with the small displacements $\delta u_i(\mathbf{r})$ from

$$\Theta_i(\mathbf{r}) = \mathrm{arctan2}(Y_i(\mathbf{r}), X_i(\mathbf{r})) \tag{S2}$$

with $i = 1, 2$ for the phase $\Theta_i(\mathbf{r})$ along x and y, respectively. These phase maps will exhibit phase slips of $\sim 2\pi$ each time the displacement $\delta u_i(\mathbf{r})$ matches a full lattice constant which we eliminate before converting the phase maps $\Theta_i(\mathbf{r})$ to the displacement field $\delta\mathbf{u}(\mathbf{r})$.

The last step is to obtain the displacement field $\delta\mathbf{u}(\mathbf{r})$ from the phase maps $\Theta_i(\mathbf{r})$. The phase $\Theta_i(\mathbf{r})$ is equal to

$$\Theta_i(\mathbf{r}) = \mathbf{q_i} \cdot \delta\mathbf{u}(\mathbf{r}) \tag{S3}$$

and thus, the displacement field $\delta\mathbf{u}(\mathbf{r})$ is obtained by inverting this linear equation. Knowing the displacement field $\delta\mathbf{u}(\mathbf{r})$, we can map the distorted lattice seen in $z_{\mathrm{m}}(\mathbf{r})$ onto a perfect lattice in $z(\mathbf{r})$.

B. Processing of QPI data

Close to the energy of the van Hove singularities, the quasi-particle interference is dominated by small QPI vectors $|\mathbf{q}| \ll |\mathbf{q}_{\mathrm{at}}|$, requiring spectroscopic $g(\mathbf{r}, V)$ maps to be acquired over large spatial areas with lateral size larger than several wavelengths $\lambda = \frac{2\pi}{|\mathbf{q}|}$ to ensure sufficient sampling of these long-wavelength QPI patterns. Acquisition of such large maps with sufficiently high spatial resolution to achieve atomic resolution for the drift correction discussed in section S2 A would result in prohibitively long measurement times. To circumvent this issue, we acquire $g(\mathbf{r}, V)$ maps which are undersampled, resulting in the atomic peaks being folded to smaller wave vectors due to aliasing. We discuss here how images are processed to reconstruct the full momentum space information from these undersampled maps.

For typical large area maps of lateral size $\sim$ 90nm, the $g(\mathbf{r}, V)$ maps were taken to acquire QPI with a pixel spacing larger than the one required for the atomic resolution. As a result, the atomic peaks appear folded to a lower q-value, as shown in Fig. S4a. To recover the original position of the atomic peaks, we apply an unfolding algorithm, Fig. S4b. Due to the long acquisition time ($\sim$ 96 hours), the tip drift will show up as a distortion in the real-space images. To correct for this distortion, we use a linear transformation to map the atomic peaks of the map onto the atomic peaks of a perfect lattice, Fig. S4c. Finally, the whole image is rotated, Fig. S4d, so that the atomic peaks are aligned with the horizontal and vertical axes. The images shown in Fig. 2 and Fig.

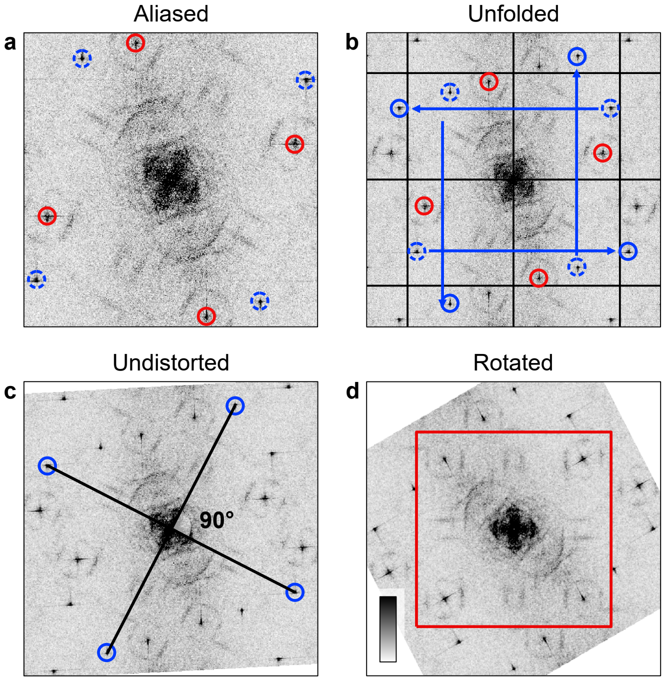

FIG. S4: **Processing of QPI data.** **a**, Fourier transform of a map layer of a differential conductance map $g_{\mathrm{m}}(\mathbf{r}, V = 1.5\mathrm{mV})$, with aliased atomic peaks indicated by the dotted blue circles. The peaks associated with the orthorhombic unit cell due to the octahedral rotations are indicated by red circles. **b**, Unfolded version of **a**, where the atomic peaks (blue dotted circles) are mapped to their correct positions. **c**, Differential conductance map $g(\mathbf{r}, V = 1.5\mathrm{mV})$ after drift correction by using a geometrical transformation to map the atomic peaks onto a perfect lattice. **d**, Rotated $g(\mathbf{r}, V)$ map to align the atomic peaks with the horizontal and vertical axes.

3 of the main manuscript were processed in this way and show the region inside the red square shown in Fig. S4d, with all relevant QPI patterns visible, but avoiding cluttering by the aliased and unfolded peaks which are outside the region of interest.

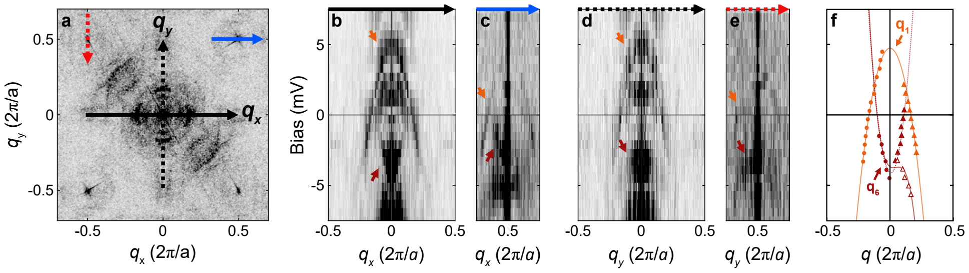

FIG. S5: **Correspondence between QPI patterns around $\mathbf{q} = (0, 0)$ and $\mathbf{q} = (\pm 0.5, 0.5)$.** **a**, QPI layer at a bias voltage of -0.3mV. The colored arrows indicate the directions along which the energy cuts shown in (**b-e**) were taken. **b**, Energy cut across $\mathbf{q} = (0, 0)$ along $\mathbf{q}_x$ (solid black arrow in **a**). **c**, Energy cut across $\mathbf{q} = (0.5, 0.5)$ along $\mathbf{q}_x$ (solid blue arrow in **a**). **d**, Energy cut across $\mathbf{q} = (0, 0)$ along $\mathbf{q}_y$ (dotted black arrow in **a**). **e**, Energy cut across $\mathbf{q} = (-0.5, 0.5)$ along $\mathbf{q}_y$ (dotted red arrow in **a**). The orange and dark red arrows indicate the dispersion corresponding to $\mathbf{q}_1$ and $\mathbf{q}_6$, respectively. **f**, **q**-vectors extracted from **b-e**, from the fit of a Lorentzian function to $\tilde{g}(\mathbf{q}, V)$ along $\mathbf{q}_x$ at each energy eV, for the dispersion of $\mathbf{q}_1$ (orange) and $\mathbf{q}_6$ (dark red). Circles show the values extracted from **b**. The solid triangles show the values extracted from **c**, around $\mathbf{q} = (0.5, 0.5)$. The open triangles correspond to the values extracted from **e**, around $\mathbf{q} = (-0.5, 0.5)$.

S3. QPI DISPERSION AROUND $\mathbf{q} = (0, 0)$ AND $\mathbf{q} = (0.5, 0.5)$

The energy layers shown in Fig. 2(f-j) of the main manuscript show QPI patterns around $\mathbf{q} = (0, 0)$ that are replicated around $\mathbf{q} = (0.5, 0.5)$, corresponding to the same scattering vectors. This is seen in Fig. S5a at -0.3mV, where the circular **q**-vector $\mathbf{q}_1$ appears around both $\mathbf{q} = (0, 0)$ and $\mathbf{q} = (0.5, 0.5)$.

The energy cuts along the arrows indicated in Fig. S5a are shown in Fig. S5(b-e), across both $\mathbf{q} = (0, 0)$ and $\mathbf{q} = (0.5, 0.5)$ and along both $\mathbf{q}_x$ and $\mathbf{q}_y$-directions. Fig. S5(b,c) show the energy cuts along $\mathbf{q}_x$, across $\mathbf{q} = (0, 0)$ and $\mathbf{q} = (0.5, 0.5)$, respectively. Both show the dispersion corresponding to $\mathbf{q}_1$ and $\mathbf{q}_6$, as indicated by the orange and dark red

arrows. Along the $\mathbf{q}_y$ direction, Fig. S5(d,e) show the dispersion corresponding to q_1 across $\mathbf{q} = (0, 0)$ and $\mathbf{q} = (0.5, 0.5)$, respectively. The dispersion of $\mathbf{q}_6$ along $\mathbf{q}_y$ shows a hole-like instead of an electron-like shape as observed along $\mathbf{q}_x$. While in Fig. S5(d), the hole-like dispersion is not easily observed, it is quite sharp in Fig. S5(e), where the $\mathbf{q}$-values can be extracted. The $\mathbf{q}$-values extracted from panels Fig. S5(b-e) are shown in Fig. S5(f). The orange circles show the $\mathbf{q}$-values extracted from Fig. S5(b), while the orange triangles were extracted from Fig. S5(c). The orange line shows a parabolic fit to the circles, which follows on top of the triangles, showing that the patterns around $\mathbf{q} = (0, 0)$ and $\mathbf{q} = (0.5, 0.5)$ correspond to the scattering between the same constant energy contours. The red circles are extracted from Fig. S5(b), showing an electron-like dispersion. The solid triangles were extracted from Fig. S5(c), while the open triangles were extracted from Fig. S5(e), showing the change from electron-like to hole-like dispersion between the $\mathbf{q}_x$ and $\mathbf{q}_y$ directions, thus revealing that $\mathbf{q}_6$ corresponds to a saddle-point van Hove singularity.

S4. EFFECTIVE MASSES ESTIMATED FROM QPI

Assuming intra-band scattering, the relative effective masses $m^\star$ of the dispersions observed in QPI can be estimated by fitting a parabolic dispersion $E(q) = \frac{\hbar^2 q^2}{8m^\star m_e} + E_0$. Table S1 shows the effective masses $m^\star$ obtained from these fits. For $\mathbf{q}_4$, the effective mass has been extracted from a linear fit to determine $\frac{\partial E(q)}{\partial q}$ and using the relation $m^\star = \frac{\hbar^2 q}{4m_e \frac{\partial E(q)}{\partial q}}$.

S5. DETERMINATION OF QUANTUM CRITICAL POINT FROM MAGNETOSTRICTION

In Fig. S6, we show the magnetostriction of $Sr_3Ru_2O_7$ as obtained via STM. These measurements were performed by ramping the field with the tip withdrawn, and then following at each field the exact same procedure to ensure that each measurement is

Along [10]/[01]				
	k_F (Å^{-1})	$\hbar v_F$ (eV · Å)	m^* (m_e)	E_0 (meV)
$\mathbf{q}_1$	0.13 ± 0.01	-0.07 ± 0.01	-14.4 ± 0.6	4.8 ± 0.3
$\mathbf{q}_4$ *	0.19 ± 0.13	-0.08 ± 0.02	-17.4 ± 4.4	-
$\mathbf{q}_5$	0.08 ± 0.01	-0.08 ± 0.01	-7.6 ± 0.6	3.5 ± 0.4
$\mathbf{q}_6$	0.09 ± 0.01	0.10 ± 0.02	6.6 ± 1.3	-4.1 ± 1.0
$\mathbf{q}_6$			(-24.8 ± 2.2)	
Along [−11]				
	k_F (Å^{-1})	$\hbar v_F$ (eV · Å)	m^* (m_e)	E_0 (meV)
$\mathbf{q}_2$	0.31 ± 0.04	-0.07 ± 0.02	-33.3 ± 5.7	11.2 ± 2.9
$\mathbf{q}_3$	0.22 ± 0.02	-0.07 ± 0.01	-22.9 ± 2.7	8.3 ± 1.5

TABLE S1: **Fermi wavevector, Fermi velocity and effective masses extracted from QPI.** Assuming the dominant scattering vectors originate from intra-band scattering, we have extracted parameters of the bandstructure from parabolic fits to the QPI data around the Fermi level. The value in the parenthesis for the effective mass of $\mathbf{q}_6$ corresponds to a parabolic fit to the hole-like dispersion along the q_x direction. * Values extracted from a linear fit as described in S4.

affected by the same systematic error due to drift of the scan piezo. To achieve this, after ramping the field we bring the STM tip back into tunneling, scanning the same $24 \times 24\text{nm}^2$ area for 30min and then measuring a topography $z(\mathbf{r})$ using the same bias set point V_{set} and current I_{set}. We then calculated the average height of the topography, $\langle z(\mathbf{r}) \rangle$, to obtain the extension of the STM scan piezo at a given magnetic field. This procedure was then repeated at different fields to obtain a measurement of the relative STM tip height as a function of field, $\delta z(\mu_0 H)$, which provides a measure of the magnetostriction of the sample [37]. We note that the measurement also contains contributions from the magnetostriction of the scan piezo, STM head and sample holder, however none of these are expected to result in sudden changes, but rather contribute a smoothly varying background.

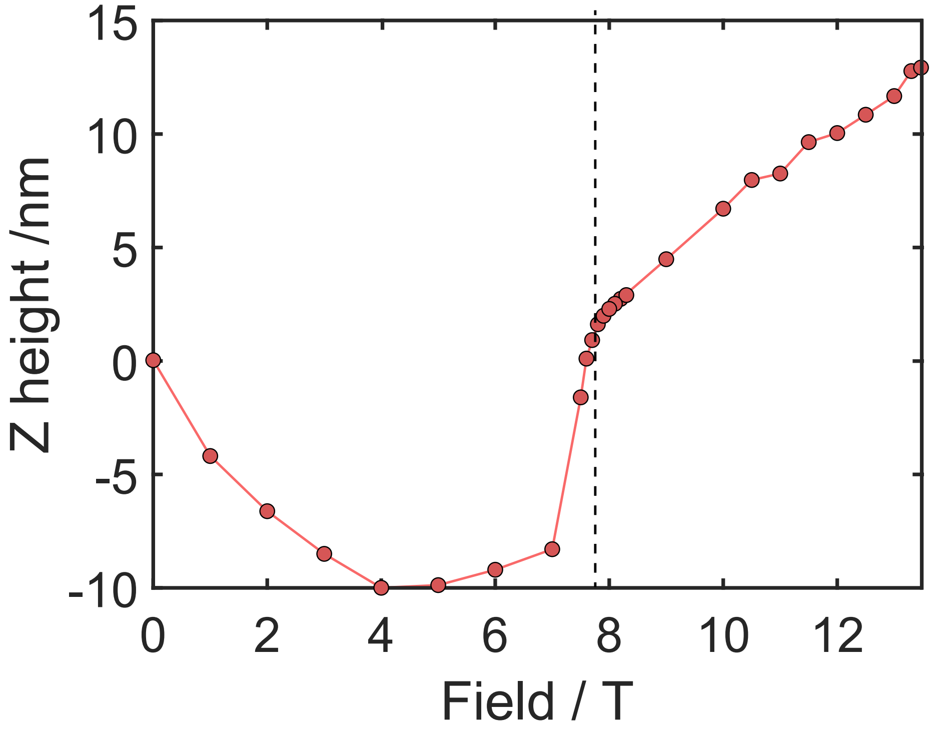

FIG. S6: **STM-based magnetostriction measurement.** Change in the average STM tip extension as a function of magnetic field. The pronounced kink is close to the magnetic field at which the bulk quantum critical point at $\mu_0 H = 7.85$T [4] is observed, indicated by the dashed vertical line ($V_{\rm set} = -5$mV, $I_{\rm set} = 91$pA).

A clear kink at $\mu_0 H = 7.85$T can be observed due to the bulk quantum critical point. The magnetostriction measurement is in good agreement with capacitive dilatometry [11].

S6. FIELD-DEPENDENCE OF POINT SPECTRA

The differential conductance spectrum shows a complex structure as a function of magnetic field, Fig. S7a, with the increase of the intensity at the Fermi level and the appearance of a high intensity feature at ~ -1.2mV. The colormap plot of $g(V, H)$ as a function of bias voltage and applied magnetic field shows an intensity shift from positive

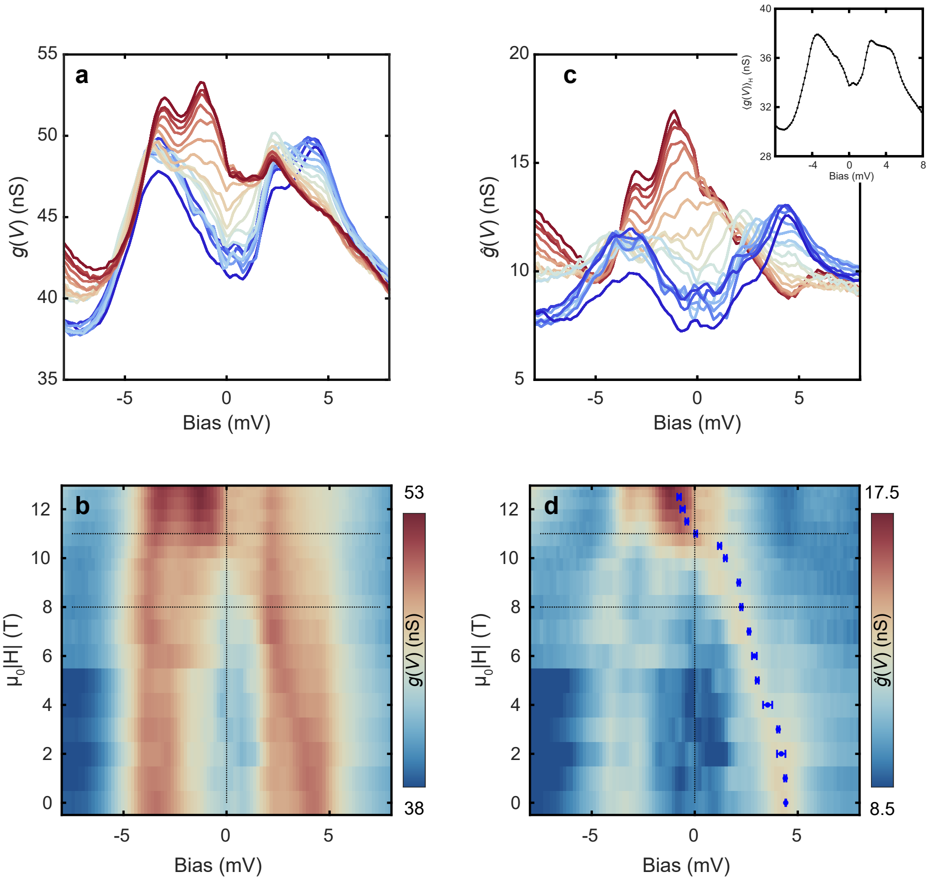

FIG. S7: **Differential conductance as function of field.** **a**, Differential conductance $g(V, H)$ as a function of magnetic field $\mu_0 H$ from $\mu_0 H = 0$T (dark blue) to 13.4T (dark red). **b**, Colormap plot of the differential conductance $g(V, H)$ as a function of bias V and magnetic field $\mu_0 H$. **c**, Differential conductance $\hat{g}(V, H)$ after subtracting the field average $\langle g(V, H)\rangle_H$ of the spectra (inset), $\hat{g}(V, H) = g(V, H) - \langle g(V, H)\rangle_H$. **d**, Colormap of $\hat{g}(V, H)$, where the shifting of a peak with magnetic field is clearly visible. The blue points show the peak positions from Lorentzian fits. The error bars correspond to the 95% confidence bounds of the fits. The dashed lines indicate 7.85T, the field of the bulk transition[4], and 11T, the field at which the peak crosses the Fermi level.

energies to negative energies, Fig. S7b. However, due to the rich structure of the $g(V,H)$ spectra, it is not easy to follow which peak is being shifted by the magnetic field. To highlight the changes in the differential conductance and determine that a peak is moving across the Fermi level, we plot the quantity $\hat{g}(V,H) = g(V,H) - \langle g(V,H) \rangle_H$, Fig. S7c, where we subtract the field average of the spectra $\langle g(V,H) \rangle_H$ (inset of Fig. S7c) to remove the field-independent background. The colormap plot of $\hat{g}(V,H)$ is shown in fig. S7d, where a peak shifting across the Fermi level can be clearly observed, reaching the Fermi level at a magnetic field of $\sim$ 11T. The $\hat{g}(V,H)$ curves make it easier to fit Lorentzian functions to find the peak positions. The blue circles in Fig. S7d represent the peak positions extracted from the Lorentzian fits, showing that the slope at which the peak moves with field decreases as it approaches the Fermi level. Fitting a linear function to all points allows for an estimation of the overall g-factor of $\sim$ 15.

S7. TEMPERATURE DEPENDENCE

We have measured spatially averaged tunneling spectra as a function of temperature T up to $T = 6$K. The data, shown in Fig. S8, shows no notable changes as a function of temperature T, any changes observed are consistent with thermal broadening.

S8. ZIGZAG ORDER IN TOPOGRAPHIC IMAGES

Fig. S9 shows the appearance of the magnetic-field-induced charge stripe order for different magnetic field strengths. The bias-dependence of the field-induced charge stripe order is shown in fig. S10. While the charge-stripe order is strongest at small bias voltages, it can be clearly detected over a wide range of bias voltages up to 100mV.

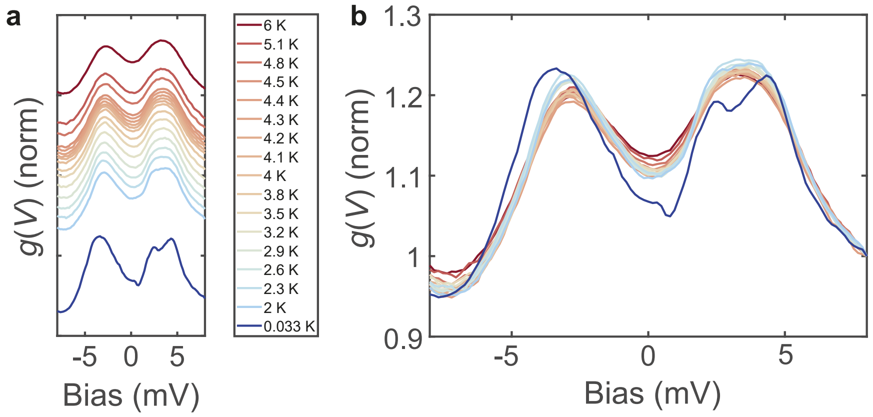

FIG. S8: **Temperature dependence of the spatially averaged tunneling conductance.** **a**, Waterfall plot of the tunneling conductance $g(V,T)$ between $T = 33$mK and 6K ($V_{\mathrm{set}} = 8$mV, $I_{\mathrm{set}} = 500$pA, $V_{\mathrm{L}} = 160\mu$V). **b**, Plot of the temperature dependent differential conductance $g(V,T)$ without offset. The change in the tunneling conductance observed here is consistent with thermal broadening. Each curve has been normalised to the differential conductance $g(V,T)$ at the maximum positive bias voltage V.

S9. DFT CALCULATIONS

DFT calculations were performed using the Quantum Espresso package [41] and projector-augmented wave pseudopotentials using the PBE exchange correlation functional. All calculations were performed on a free-standing monolayer of $Sr_3Ru_2O_7$ with 15Å of vacuum. Calculations were done with a plane-wave cut-off of 40Ry, a wavefunction cut-off of 320Ry and a **k**-grid of $7 \times 7 \times 1$. For the E-type antiferromagnetic order a supercell of $2 \times 1 \times 1$ was used with positive magnetic moment on the Ru atoms in one unit cell, and negative moment for those in the second unit cell. The resulting Fermi surfaces for a paramagnetic, ferromagnetic and E-type ordered phase are shown in fig. S11.

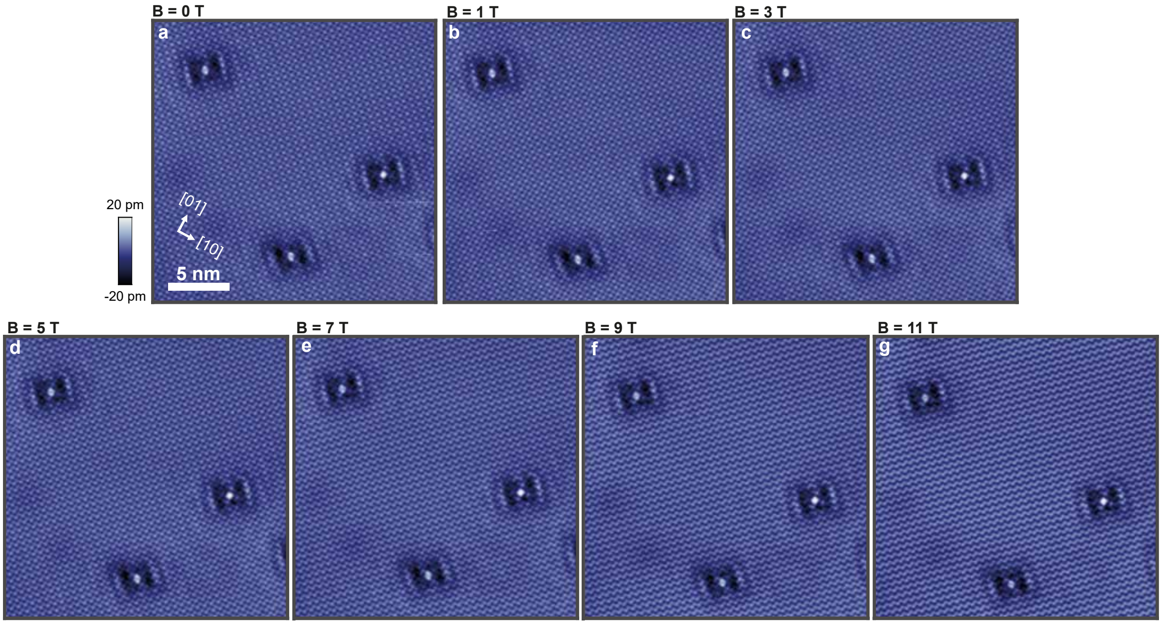

FIG. S9: **Appearance of the zigzag order with magnetic field.** Topographic images taken in a $24 \times 24\text{nm}^2$ area ($V_{\rm set} = -5$mV, $I_{\rm set} = 91$pA) for **a** $\mu_0 H = 0$T, **b** 1T, **c** 3T, **d** 5T, **e** 7T, **f** 9T, **g** 11T.

S10. TI-SUBSTITUTED $SR_3RU_2O_7$

Previous quasi-particle interference measurements have been carried out on Ti-doped samples[22]. Here, we show QPI measurements on a $Sr_3(Ru_{0.99}Ti_{0.01})_2O_7$ sample, i.e. with 1% Ti doping as used also by Lee et al.[22], demonstrating that the main C_2-symmetry breaking feature reported in the main text remains the same also with an increased concentration of scatterers. Fig. S12 shows a topographic image, QPI map and tunneling spectra obtained from the Ti-doped sample. The topographic image (Fig. S12a) shows the Ti defects as dark depressions, in addition to the defects found in clean samples. As in the clean samples, clear C_2-symmetry breaking scattering patterns are observed around some defects, as also confirmed by the Fourier transformation of a conductance map (Fig. S12b). The most prominent difference to undoped samples is seen in the tunneling

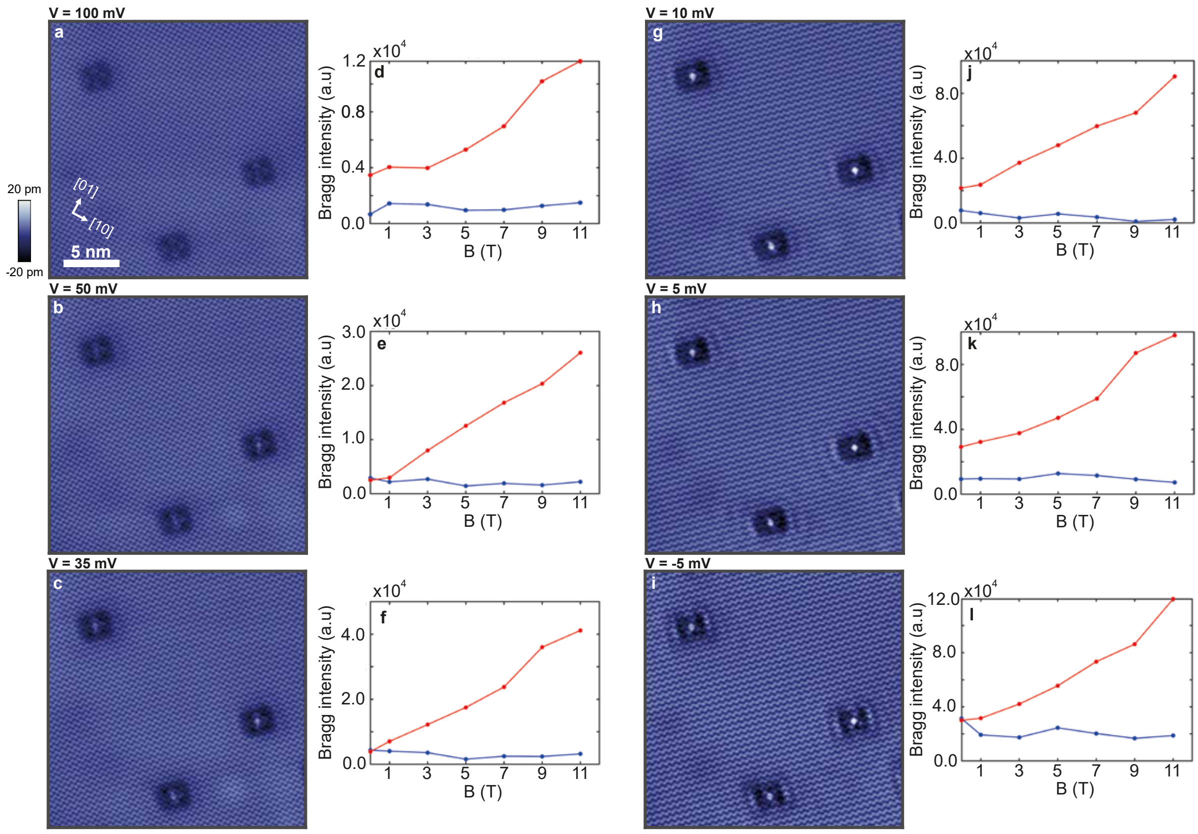

FIG. S10: **Bias dependence of the zigzag order. a-c, g-i** Topographies $z(\mathbf{r})$ measured at a magnetic field $\mu_0 H = 11$T using different bias voltages V_{set}, the corresponding graphs to the right (**d-f, j-l**) represent the intensity of the Fourier peaks associated with the charge order, as discussed in the main text. **a, d** $V_{\text{set}} = 100$mV; **b, e** $V_{\text{set}} = 50$mV; **c, f** $V_{\text{set}} = 35$mV; **g, j** $V_{\text{set}} = 10$mV; **h, k** $V_{\text{set}} = 5$mV; **i, l** $V_{\text{set}} = -5$mV. The current I_{set} was set such that the junction resistance R_{set} remained constant with $R_{\text{set}} = 55\text{M}\Omega$.

spectra, where the double-peak structure around the Fermi energy is suppressed, leaving behind only a gap-like feature at the Fermi energy (Fig. S12c, d). Interestingly, though, the additional disorder does not destroy the C_2 symmetry breaking, as might have been suggested by the sensitivity of the nematic phase to defects found in the bulk[1].

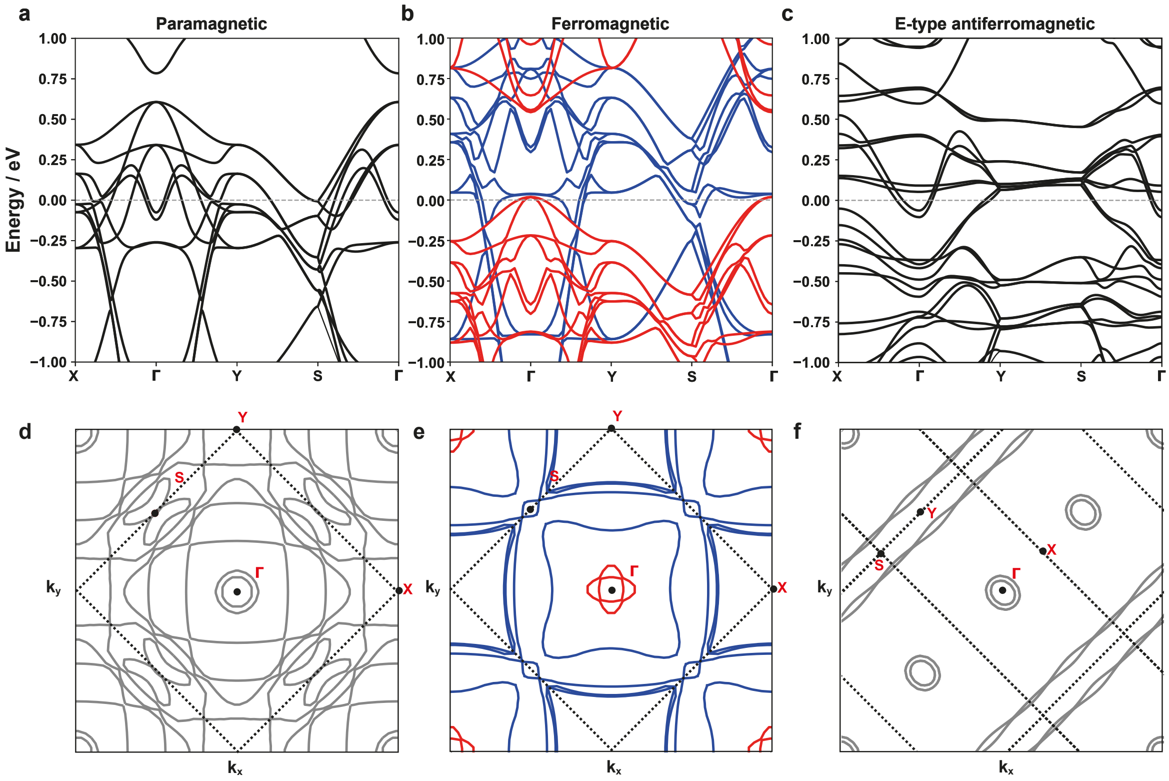

FIG. S11: **DFT study of the electronic structure for different magnetic states in $\mathbf{Sr_3Ru_2O_7}$. a-c**, The low energy band structure for **a** the paramagnetic state, **b** the ferromagnetic state and **c** E-type antiferromagnetic order. **d-f**, corresponding Fermi surfaces.

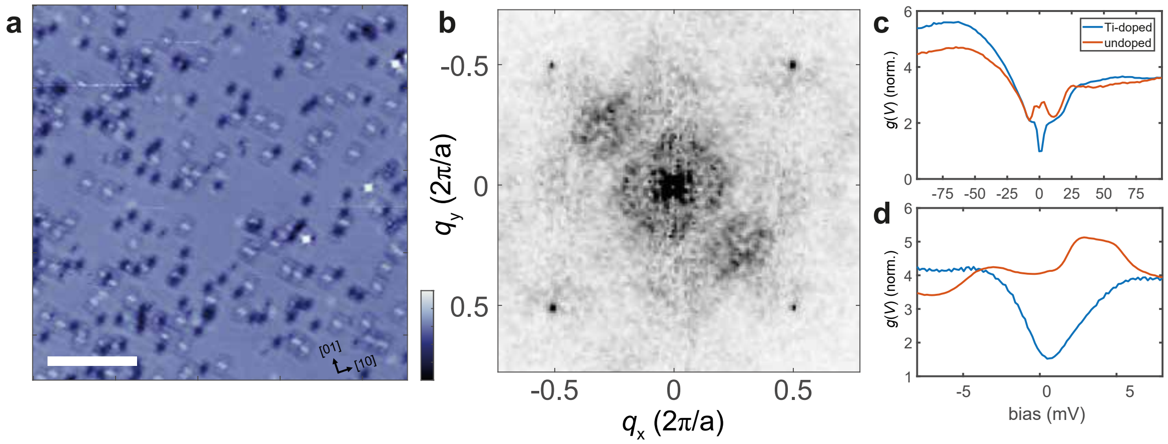

FIG. S12: **Comparison with Ti-doped $Sr_3Ru_2O_7$.** **a**, Topographic image of the surface of a Ti-doped sample of $Sr_3Ru_2O_7$. The dark spots are Ti defects. The defects showing the C_2-symmetric scattering patterns seen in the undoped sample can still be observed here (scale bar: 10nm, $V_{\rm set} = 10$mV, $I_{\rm set} = 100$pA). **b**, Fourier transform of a spectroscopic map at $V = -2$mV. The dominant C_2 symmetric scattering vector can still be clearly seen ($V_{\rm set} = 10$mV, $I_{\rm set} = 100$pA, $V_{\rm L} = 0.5$mV). **c**, Tunneling spectrum on the Ti-doped sample (blue) at $T = 2.1$K and the undoped sample (orange) at $T = 80$mK ($V_{\rm set} = 100$mV, $I_{\rm set} = 250$pA, $V_{\rm L} = 1.9$mV). **d**, Comparison of a spatially averaged tunneling spectrum of the Ti-doped (blue) and undoped (orange) sample, measured at $T = 2$K ($V_{\rm set} = 8$mV, $I_{\rm set} = 500$pA, $V_{\rm L} = 0.16$mV. The spectrum recorded on the Ti-doped sample was normalised with respect to the undoped sample at the maximum bias value.

REFERENCES AND NOTES

1. R. A. Borzi, S. A. Grigera, J. Farrell, R. S. Perry, S. J. S. Lister, S. L. Lee, D. A. Tennant, Y. Maeno, A. P. Mackenzie, Formation of a nematic fluid at high fields in $Sr_3Ru_2O_7$. *Science* **315**, 214–217 (2007).

2. P. Gegenwart, Q. Si, F. Steglich, Quantum criticality in heavy-fermion metals. *Nat. Phys.* **4**, 186–197 (2008).

3. S. Sachdev, B. Keimer, Quantum criticality. *Phys. Today* **64**, 29–35 (2011).

4. S. A. Grigera, R S Perry, A. J. Schofield, M. Chiao, S. R. Julian, G G Lonzarich, S. I. Ikeda, Y. Maeno, A. J. Millis, A P Mackenzie, Magnetic field-tuned quantum criticality in the metallic ruthenate $Sr_3Ru_2O_7$. *Science* **294**, 329–332 (2001).

5. A. W. Rost, R. S. Perry, J.-F. Mercure, A. P. Mackenzie, S. A. Grigera, Entropy landscape of phase formation associated with quantum criticality in $Sr_3Ru_2O_7$. *Science*, **325**, 1360–1363 (2009).

6. A. W. Rost, A. M. Berridge, R. S. Perry, J.-F. Mercure, S. A. Grigera, A. P. Mackenzie, Power law specfic heat divergence in $Sr_3Ru_2O_7$. *Phys. Stat. Sol. B* **247**, 513–515 (2010).

7. Y. Tokiwa, M. Mchalwat, R. S. Perry, P. Gegenwart, Multiple metamagnetic quantum criticality in $Sr_3Ru_2O_7$. *Phys. Rev. Lett.* **116**, 226402 (2016).

8. D. Sun, A. W. Rost, R. S. Perry, A. P. Mackenzie, M. Brando, Low temperature thermodynamic investigation of the phase diagram of $Sr_3Ru_2O_7$. *Phys. Rev. B* **97**, 115101 (2018).

9. D. V. Efremov, A. Shtyk, A. W. Rost, C. Chamon, A. P. Mackenzie, J. J. Betouras, Multicritical fermi surface topological transitions. *Phys. Rev. Lett.* **123**, 207202 (2019).

10. C. Lester, S. Ramos, R. S. Perry, T. P. Croft, R. I. Bewley, T. Guidi, P. Manuel, D. D. Khalyavin, E. M. Forgan, S. M. Hayden, Field-tunable spin-density-wave phases in $Sr_3Ru_2O_7$. *Nat. Mater.* **14**, 373–378 (2015).

11. P. Gegenwart, F. Weickert, R. S. Perry, Y. Maeno, Low-temperature magnetostriction of $Sr_3Ru_2O_7$. *Phys. B Condens. Matter* **378-380**, 117–118 (2006).

12. R. S. Perry, L. M. Galvin, S. A. Grigera, L. Capogna, A. J. Schofield, A. P. Mackenzie, M. Chiao, S. R. Julian, S. I. Ikeda, S. Nakatsuji, Y. Maeno, C. Pfleiderer. Metamagnetism and critical fluctuations in high quality single crystals of the bilayer ruthenate $Sr_3Ru_2O_7$. *Phys. Rev. Lett.* **86**, 2661–2664 (2001).

13. B. Binz, M. Sigrist, Metamagnetism of itinerant electrons in multi-layer ruthenates. *Europhys. Lett.* **65**, 816–822 (2004).

14. A. Hackl, M. Vojta, Zeeman-driven lifshitz transition: A model for the experimentally observed fermi-surface reconstruction in $YbRh_2Si_2$. *Phys. Rev. Lett.* **106**, 137002 (2011).

15. W.-C. Lee, C. Wu, Theory of unconventional metamagnetic electron states in orbital band systems. *Phys. Rev. B* **80**, 104438 (2009).

16. S. Raghu, A. Paramekanti, E A. Kim, R. A. Borzi, S. A. Grigera, A. P. Mackenzie, S. A. Kivelson, Microscopic theory of the nematic phase in $Sr_3Ru_2O_7$. *Phys. Rev. B* **79**, 214402 (2009).

17. C. M. Puetter, J. G. Rau, H.-Y. Kee, Microscopic route to nematicity in $Sr_3Ru_2O_7$. *Phys. Rev. B* **81**, 081105 (2010).

18. W.-C. Lee, D. P. Arovas, C. Wu, Quasiparticle interference in the unconventional metamagnetic compound $Sr_3Ru_2O_7$. *Phys. Rev. B* **81**, 184403 (2010).

19. C. A. Marques, L. C. Rhodes, R. Fittipaldi, V. Granata, C.-M. Yim, R. Buzio, A. Gerbi, A. Vecchione, A. W. Rost, P. Wahl. Magnetic-field tunable intertwined checkerboard charge order and nematicity in the surface layer of Sr_2RuO_4. *Adv. Mater.* **33**, 2100593 (2021).

20. H. Shaked, J. D. Jorgensen, O. Chmaissem, S. Ikeda, Y. Maeno. Neutron diffraction study of the structural distortions in $Sr_3Ru_2O_7$. *J. Solid State Chem.* **154**, 361–367 (2000).

21. M. Behrmann, C. Piefke, F. Lechermann, Multiorbital physics in Fermi liquids prone to magnetic order. *Phys. Rev. B* **86**, 045130 (2012).

22. J. Lee, M. P. Allan, M. A. Wang, J. Farrell, S. A. Grigera, F. Baumberger, J. C. Davis, A. P. Mackenzie, Heavy d-electron quasiparticle interference and real-space electronic structure of $Sr_3Ru_2O_7$. *Nat. Phys.* **5**, 800–804 (2009).

23. K. Iwaya, S. Satow, T. Hanaguri, N. Shannon, Y. Yoshida, S. I. Ikeda, J. P. He, Y. Kaneko, Y. Tokura, T. Yamada, H. Takagi, Local tunneling spectroscopy across a metamagnetic critical point in the bilayer ruthenate $Sr_3Ru_2O_7$. *Phys. Rev. Lett.* **99**, 057208 (2007).

24. A. Tamai, M. P. Allan, J. F. Mercure, W. Meevasana, R. Dunkel, D. H. Lu, R. S. Perry, A. P. Mackenzie, D. J. Singh, Z.-X. Shen, F. Baumberger, Fermi surface and van Hove singularities in the itinerant metamagnet $Sr_3Ru_2O_7$. *Phys. Rev. Lett.* **101**, 026407 (2008).

25. L. C. Rhodes, M. D. Watson, T. K. Kim, M. Eschrig, k_z selective scattering within quasiparticle interference measurements of FeSe. *Phys. Rev. Lett.* **123**, 216404 (2019).

26. C. A. Marques, M. S. Bahramy, C. Trainer, I. Markovic, M. D. Watson, F. Mazzola, A. Rajan, T. D. Raub, P. D. C. King, P. Wahl, Tomographic mapping of the hidden dimension in quasi-particle interference. *Nat. Commun.* **12**, 6739 (2021).

27. J.-F. Mercure, A. W. Rost, E. C. T. O'Farrell, S. K. Goh, R. S. Perry, M. L. Sutherland, S. A. Grigera, R. A. Borzi, P. Gegenwart, A. S. Gibbs, A. P. Mackenzie. Quantum oscillations near the metamagnetic transition in $Sr_3Ru_2O_7$. *Phys. Rev. B* **81**, 235103 (2010).

28. B. Hu, G. T. McCandless, M. Menard, V. B. Nascimento, J. Y. Chan, E. W. Plummer, R. Jin, Surface and bulk structural properties of single-crystalline $Sr_3Ru_2O_7$. *Phys. Rev. B* **81**, 184104 (2010).

29. S.-I. Ikeda, N. Shirakawa, T. Yanagisawa, Y. Yoshida, S. Koikegami, S. Koike, M. Y. Uwatoko, Uniaxial-pressure induced ferromagnetism of enhanced paramagnetic $Sr_3Ru_2O_7$. *J. Physical Soc. Japan* **73**, 1322–1325 (2004).

30. W. Wu, A. McCollam, S. A. Grigera, R. S. Perry, A. P. Mackenzie, S. R. Julian. Quantum critical metamagnetism of $Sr_3Ru_2O_7$ under hydrostatic pressure. *Phys. Rev. B* **83**, 045106 (2011).

31. D. O. Brodsky, M. E. Barber, J. A. N. Bruin, R. A. Borzi, S. A. Grigera, R. S. Perry, A. P. Mackenzie, C. W. Hicks, Strain and vector magnetic field tuning of the anomalous phase in $Sr_3Ru_2O_7$. *Sci. Adv.* **3**, e1501804 (2017).

32. A. Putatunda, G. Qin, W. Ren, D. J. Singh. Competing magnetic orders in quantum critical $Sr_3Ru_2O_7$. *Phys. Rev. B* **102**, 014442 (2020).

33. M. Zhu, Y. Wang, P. G. Li, J. J. Ge, W. Tian, D. Keavney, Z. Q. Mao, X. Ke. Tipping the magnetic instability in paramagnetic $Sr_3Ru_2O_7$ by Fe impurities. *Phys. Rev. B* **95**, 174430 (2017).

34. D. Mesa, F. Ye, S. Chi, J. A. Fernandez-Baca, W. Tian, B. Hu, R. Jin, E. W. Plummer, J. Zhang, Single-bilayer *E*-type antiferromagnetism in Mn-substituted $Sr_3Ru_2O_7$: Neutron scattering study. *Phys. Rev. B* **85**, 180410 (2012).

35. P. Steffens, J. Farrell, S. Price, A. P. Mackenzie, Y. Sidis, K. Schmalzl, M. Braden, Incommensurate magnetic ordering in Ti-doped $Sr_3Ru_2O_7$. *Phys. Rev. B* **79**, 054422 (2009).

36. R. Fittipaldi, R. Hartmann, M. T. Mercaldo, S. Komori, A. Bjørlig, W. Kyung, Y. Yasui, T. Miyoshi, L. A. B. Olde Olthof, C. M. Palomares Garcia, V. Granata, I. Keren, W. Higemoto, A. Suter, T. Prokscha, A. Romano, C. Noce, C. Kim, Y. Maeno, E. Scheer, B. Kalisky, J. W. A. Robinson, M. Cuoco, Z. Salman, A. Vecchione, A. Di Bernardo. Unveiling unconventional magnetism at the surface of Sr_2RuO_4. *Nat. Commun.* **12**, 5792 (2021).

37. C. Trainer, C. Abel, S. L. Bud'ko, P. C. Caneld, P. Wahl, Phase diagram of Ce Sb_2 from magnetostriction and magnetization measurements: Evidence for ferrimagnetic and antiferromagnetic states. *Phys. Rev. B* **104**, 205134 (2021).

38. U. R. Singh, M. Enayat, S. C. White, P. Wahl, Construction and performance of a dilution-refrigerator based spectroscopic-imaging scanning tunneling microscope. *Rev. Sci. Instrum.* **84**, 013708 (2013).

39. C. A. Marques, M. J. Neat, C.-M. Yim, M. D. Watson, L. C. Rhodes, C. Heil, K. Pervakov, V. Vlasenko, V. Pudalov, A. Muratov, T. Kim, P. Wahl, Electronic structure and superconductivity of the non-centrosymmetric Sn_4As_3. *New J. Phys.* **22**, 063049 (2020).

40. M. J. Lawler, K. Fujita, J. Lee, A. R. Schmidt, Y. Kohsaka, C.-K. Kim, H. Eisaki, S. Uchida, J. C. Davis, J. P. Sethna, E.-A. Kim, Intra-unit-cell electronic nematicity of the high-T_c copper-oxide pseudogap states. *Nature* **466**, 347–351 (2010).

41. P. Giannozzi, O. Baseggio, P. Bonfa, D. Brunato, R. Car, I. Carnimeo, C. Cavazzoni, S. de Gironcoli, P. Delugas, F. Ferrari Runo, A. Ferretti, N. Marzari, I. Timrov, A. Urru, S. Baroni. Quantum espresso toward the exascale. *J. Chem. Phys.* **152**, 154105 (2020).
